# Restoring blue carbon ecosystems

**DOI:** 10.1017/cft.2024.9

**Published:** 2024-05-17

**Authors:** Daniel A. Friess, Zoë I. Shribman, Milica Stankovic, Naima Iram, Melissa M. Baustian, Carolyn J. Ewers Lewis

**Affiliations:** 1 Department of Earth and Environmental Sciences, Tulane University, New Orleans, LA, USA; 2 Excellence Center for Biodiversity of Peninsular Thailand, Faculty of Science, Prince of Songkla University, Songkhla, Thailand; 3 Centre for Nature-Based Climate Solutions, National University of Singapore, Singapore, Singapore; 4 U.S. Geological Survey, Wetland and Aquatic Research Center, Baton Rouge, LA, USA; 5 Department of Natural Sciences, Flagler College, Saint Augustine, FL, USA

**Keywords:** mangrove, marsh, natural climate solution, rehabilitation, seagrass

## Abstract

Mangroves, tidal marshes and seagrasses have experienced extensive historical reduction in extent due to direct and indirect effects of anthropogenic land use change. Habitat loss has contributed carbon emissions and led to foregone opportunities for carbon sequestration, which are disproportionately large due to high ‘blue carbon’ stocks and sequestration rates in these coastal ecosystems. As such, there has been a rapid increase in interest in using coastal habitat restoration as a climate change mitigation tool. This review shows that restoration efforts are able to substantially increase blue carbon stocks, while also having a positive impact on various gaseous fluxes. However, blue carbon increases are spatially variable, due to biophysical factors such as climate and geomorphic setting. While there are potentially hundreds of thousands of hectares of land that may be biophysically suitable for restoration, these activities are still often conducted at small scales and with mixed success. Maximizing potential carbon gains through blue carbon restoration will require managers and coastal planners to overcome the myriad socioeconomic and governance constraints related to land tenure, legislation, target setting and cost, which often push restoration projects into locations that are biophysically unsuitable for plant colonization.

## Impact statement

Coastal habitats such as mangroves, tidal marshes and seagrasses have faced extensive loss due to human activities such as land use change and pollution. This loss occurs despite the value of these ecosystems as important reservoirs of ‘blue carbon’, with much of their carbon stored in the soil column. Recent interest has turned to blue carbon conservation and restoration, with managers and policy makers around the world setting targets to restore habitats to generate carbon credits or meet national targets for climate change mitigation that are larger in magnitude than targets previously set. This article collates the empirical evidence base for how restoration activities can positively impact various parts of the blue carbon cycle and contribute to climate change mitigation. However, carbon benefits will only happen if projects can overcome various socioeconomic, governance and biophysical constraints to restoration that currently limit our ability to restore coastal landscapes at the scale required to tackle the climate change challenge.

## Introduction

Natural climate solutions – actions that protect, sustainably manage and restore ecosystems – are an important tool in mitigating climate change and keeping global temperatures below a 2 °C increase by the end of this century. Natural climate solutions, such as those provided by forests, wetlands, grasslands and agricultural lands, could provide one-third of the cost-effective climate mitigation needed to achieve this goal, equivalent to 23.8 Pg CO_2_ yr.^−1^ (Griscom et al., 2017), while providing a wide range of co-benefits, such as food provision, livelihoods and cultural services to local communities.

A natural climate solution that has gained substantial interest over the last decade is the conservation and restoration of blue carbon. The current definition of blue carbon genereally refers to the carbon sequestered and stored in three specific coastal ecosystems: mangroves, tidal marshes and seagrasses. Mangroves are a community of salt-tolerant trees covering >145,000 km^2^ (Jia et al., 2023) in the tropics, subtropics and warm temperate regions. In some instances, they are able to store almost 2,800 Mg C ha^−1^ in soil layers 1–6 m deep (Adame et al., 2021). Tidal marshes cover 53,000 km^2^ globally (Worthington et al., 2024), while estimates of global seagrass extent vary from ~160,000 km^2^ (McKenzie et al., 2020) to ~1.65 million km^2^ (Jayathilake and Costello, 2018), depending on the methods used for mapping these systems. Together, these three ecosystems store >30,000 Tg C (Macreadie et al., 2021).

Despite their importance for climate change mitigation, blue carbon ecosystems have experienced substantial declines in area due to human land use change and coastline modification, and continue to be lost around the world (Fluet-Chouinard et al., 2023). Mangroves were coarsely estimated to be lost at rates of 1–3% yr.^−1^ in the 20th century, although this has reduced to approximately 0.1–0.2% from 2000 onward (Friess et al., 2019). Tidal marshes have been lost at 0.28% yr.^−1^ in the 21st century (Campbell et al., 2022), and seagrasses are threatened across much of their global range, with losses of at least 5,602 km^2^ since 1880 (Dunic et al., 2021). Habitat loss contributes to climate change through the emissions of stored carbon; in the 21st century, global mangrove loss led to the emissions of 26.3 Tg CO_2_e yr.^−1^ (Hamilton and Friess, 2018), while tidal marsh loss released 16.3 Tg CO_2_e yr.^−1^ to the atmosphere (Campbell et al., 2022).

Lost areas of habitat provide an opportunity for new blue carbon accumulation through habitat restoration. In coastal wetlands, the term ‘restoration’ encompasses a range of management actions. They can generally be defined as the planting of seedlings (*mangroves*, Zimmer et al., 2022; *tidal marshes*, Sparks et al., 2013; *seagrasses*, van Katwijk et al., 2016) or the encouragement of natural regeneration, often through the removal of environmental stressors or the reintroduction of hydrological flows (*mangroves*, Lewis, 2005; *tidal marshes*, Garbutt and Wolters, 2008; *seagrasses, Bourderesque et al., 2021*) and/or the broadcasting of seeds and propagules on high tides (e.g., Orth et al., 2020). Coastal restoration activities are attracting substantial recent interest and funding (UNEP-WCMC, 2022), and restoration is a management action that is now the basis of several blue carbon projects (Friess et al., 2022a).

In this review, we synthesize the experiences of the restoration of mangroves, tidal marshes and seagrasses for blue carbon outcomes. Specifically, we outline: (i) the global-scale biophysical potential for the restoration of blue carbon ecosystems; (ii) estimates of blue carbon benefits following coastal habitat restoration; and (iii) current challenges and constraints to the effective restoration of blue carbon ecosystems. We aim for this to be a broad synthesis of the potential benefits of restoration and challenges to their implementation, and we refer the reader to in-depth or systematic reviews on specific topics where appropriate.

## Large-scale scope of restoration for blue carbon

Due to centuries of coastal habitat loss, a large extent globally of areas could be reverted to their original state, with concomitant gains in blue carbon. Macreadie et al. (2021) estimate this area to be approximately 30 million ha (with a 95% confidence interval of 17.5–41.6 million ha), with seagrasses accounting for 57% and mangroves accounting for 37% of this potential. Tidal marshes have the lowest global restoration potential, probably because current land uses (such as urban development in estuaries) preclude conversion back to tidal marsh. If the restoration of blue carbon ecosystems could be conducted successfully at this scale, it could result in the removal of 841 Tg CO_2_e per year from the atmosphere, equivalent to ~3% of global fossil fuel emissions (Macreadie et al., 2021). Studies for mangroves specifically suggest that 8,120 km^2^ deforested since 1996 might be biophysically restorable (Worthington and Spalding, 2018); resulting in 365 million tonnes of carbon gains once fully restored.

Such large potential carbon gains align with several international initiatives promoting large-scale coastal habitat restoration, such as the Trillion Trees Initiative and the Bonn Challenge (Lovelock et al., 2022), although interest and implementation from these initiatives in blue carbon ecosystems could still increase further to match that of terrestrial ecosystems (Waltham et al., 2020). Countries have also identified large-scale opportunities for blue carbon restoration. For example, Indonesia has a target of restoring 600,000 ha of mangroves by 2024, with carbon gains as a key driver of this policy (Sidik et al., 2023). While studies suggest that only ~186,600 ha (Worthington and Spalding, 2018) to 193,367 ha (Sasmito et al., 2023) are biophysically suitable for restoration in Indonesia, it shows the commitment that some countries have for blue carbon restoration. Other countries have set blue carbon restoration targets within their Nationally Determined Contributions to the Paris Agreement by 2030; in 2020–2021, Belize committed to restoring <4,000 ha of mangroves, Haiti committed to increasing its mangrove area to 19,500 ha and Samoa committed to increasing its mangrove area by 5% (Friess, 2023).

## Carbon benefits of restoration

**Figure 1.**
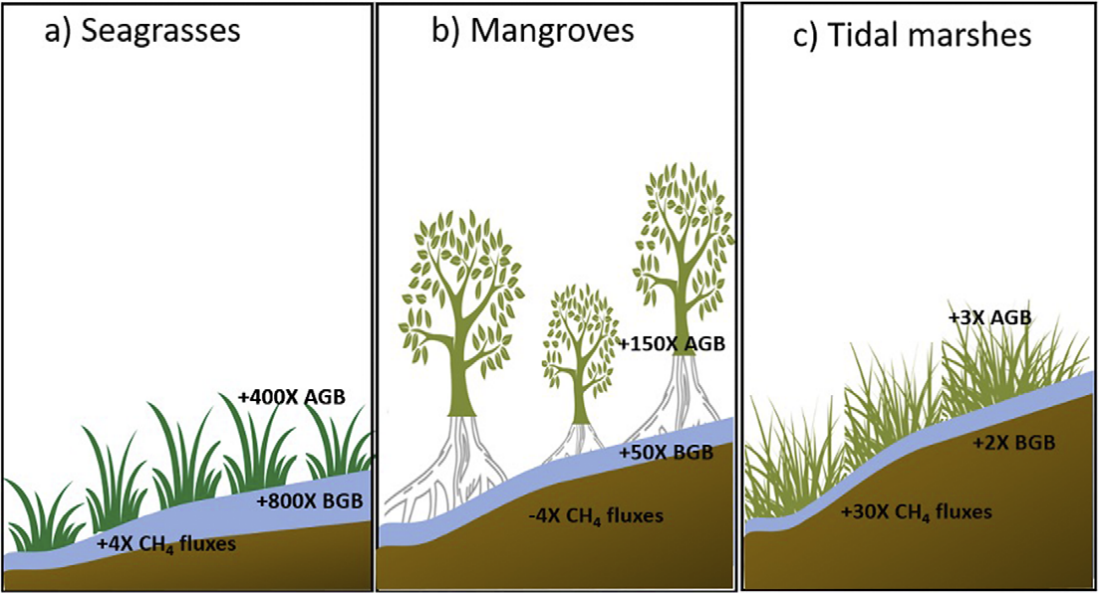
Restored blue carbon stocks (aboveground and belowground, AGB and BGB) tend to be 2–800 times higher than in degraded/converted/bare sites, and methane (CH_4_) fluxes can be four times less than degraded/converted/bare sites, depending on habitats and age. For example, (a) seagrasses, bare versus (vs) restored (Oreska et al., 2020); (b) Mangrove, converted/degraded vs restored (Sasmito et al., 2019; Rosentreter et al., 2021) and (c) tidal marshes; converted/degraded vs restored (Stagg and Mendelssohn, 2010; Iram et al., 2022; Shao et al., 2022; Kelsall et al., 2023); refer to the original references for details; positive values indicate an increase and negative values indicate a decrease.

The achievement of meaningful coastal restoration targets is expected to have important impacts on blue carbon cycling and storage (Figure 1). Restoration will have positive impacts on aboveground and belowground biomass pools, with carbon stocks increasing by 2–800 times, depending on the ecosystem and setting (Stagg and Mendelssohn, 2010; Sasmito et al., 2019; Oreska et al., 2020; Rosentreter et al., 2021; Iram et al., 2022; Shao et al., 2022; Kelsall et al., 2023). Similar positive impacts are expected on the soil carbon pool (generally referring to the top 1 m of sediment), although patterns are more mixed due to legacy carbon from prior land uses. Positive impacts on carbon fluxes are generally expected. For example, methane emissions would be expected to be as much as four times lower than degraded sites or those under other land uses, particularly if the replaced habitat was dominated by freshwater, due to the influence of salinity on methanogenesis (e.g., Cotavicz et al., 2024).

The following describes major stock and flux components separately for each blue carbon ecosystem, and is indicative of the magnitude of stock and flux change that can be expected following restoration. For in-depth reviews of general carbon stocks and fluxes for different blue carbon ecosystems, we direct the reader to systematic reviews such as Fourqurean et al. (2012) for seagrass stocks; Johannessen (2022) for seagrass carbon accumulation rates; Sasmito et al. (2019) for carbon stock change and GHG flux change with mangrove restoration; Mason et al. (2023) for carbon accumulation rates and GHG fluxes for tidal marshes; and Taillardat et al. (2020) for GHG fluxes for multiple blue carbon ecosystems. For further information on geomorphic and ecological drivers of blue carbon stocks and fluxes, we direct the reader to a recent review by Kirwan et al. (2023).

### Mangroves

Mangrove restoration efforts vary in the degree of human intervention, and include ecological engineering, monoculture plantations, afforestation and ecological mangrove restoration that promotes natural regeneration (Ellison et al., 2020). The latter is encouraged through hydrological modification, including the construction or reconfiguration of tidal creeks, culverts, sediment additions to change elevation and tidal reintroduction (Lewis, 2005). Restoration projects are increasingly incorporating carbon benefits, and chronosequence studies are being used to understand how blue carbon dynamics change with restoration age (Osland et al., 2012; Andreetta et al., 2016; Marchand, 2017; Walcker et al., 2018; Wang et al., 2021; Azman et al., 2023) alongside systematic literature reviews (Sasmito et al., 2019; Rivera-Monroy et al., 2022) to estimate carbon outcomes from restoration.

Total ecosystem carbon stocks in restored mangroves generally increase with site age. For example, studies in southeast Australia indicated a strong relationship between carbon and site age (Carnell et al., 2022), with stocks in older restored forests (17 and 35 years) averaging ~115 MgC ha^−1^, compared to only ~50 MgC ha^−1^ in younger forests (13 years) (Carnell et al., 2022). In a restoration chronosequence study in Vietnam, total ecosystem carbon stocks also increased with stand age (2 to 27 years old) from ~201 to ~519 MgC ha^−1^ (Pham et al., 2017). However, specific carbon pools may respond differently to restoration; meta-analyses suggest that mangrove biomass carbon increases for 15 years at a rate of 4 MgC ha^−1^ yr.^−1^ after restoration, although mixed patterns are observed in soil carbon stocks up to 1 m depth (Sasmito et al., 2019).

Although aboveground biomass can regenerate rapidly, carbon storage belowground takes substantially longer to replenish, with shallow soil carbon pools restocked more quickly than deeper layers due to organic matter and allochthonous inputs. A study in Thailand of hydraulic restoration and planting following conversion to shrimp ponds reported that soil organic carbon concentration increased over 4 years and stocks increased from 110 to 160 MgC ha^−1^ in 2 years, based on measurements at a 5 cm soil depth (Matsui et al., 2010). In created mangroves along a 20-year chronosequence in southwest Florida, soil organic matter and total carbon increased with site age in the upper 10 cm of the soil and were estimated to need 18–28 years to reach natural equivalence (Osland et al., 2012). Total carbon in the upper 10 cm soil layer was 30 g kg^−1^ in the created wetland compared to 144 g kg^−1^ in the natural wetland, and 13 g kg^−1^ and 88 g kg^−1^ in the lower 10–30 cm layer in the created and natural wetlands (Osland et al., 2012). Other studies have observed that soil organic carbon increases with restoration activities (Zhang et al., 2012; Dung et al., 2016; Pham et al., 2017; Sasmito et al., 2019; Ratul et al., 2022; Thura et al., 2023). Restoration activity can also influence soil carbon returns; a global review found that restoration was more effective at accumulating carbon in the top meter of soil over 40 years compared to afforestation projects (Song et al., 2023). In some cases, carbon stocks in restored mangroves can exceed those in natural systems, but differences in these comparisons may be attributed to local hydrogeomorphological controls on carbon storage (Kusumaningtyas et al., 2022).

While most research has focused on changes in carbon stocks over time, we lack a clear understanding of how carbon sequestration and greenhouse gas fluxes respond to mangrove restoration. Limited evidence from southeast Australia showed that sequestration rates do respond to site age, at ~3 Mg C ha^−1^ yr.^−1^ in older forests compared to ~1.5 Mg C ha^−1^ yr.^−1^ in younger forests (Carnell et al., 2022). Methane fluxes decreased with age, being ~5 times higher in the younger forest compared to older forests, with rates of 5.8 mg CH_4_ m^−2^ day^−1^ (Carnell et al., 2022). Flux measurements are important to understand the climate change mitigation potential of mangrove restoration; in an analysis of mangrove restoration offset potential in North Sulawesi, Indonesia, carbon mitigation was estimated at −27.6 Mg CO_2_-e ha^−1^ yr.^−1^ (Cameron et al., 2019). Scaled up to the national level in Indonesia, this could represent an offset potential of up to 16.56 million Mg CO_2_-e ha^−1^ yr.^−1^ (Cameron et al., 2019).

### Tidal marshes

There is global interest in restoring tidal marshes to recover lost ecosystem functions and services (Macreadie et al., 2017; Kelleway et al., 2020; Hagger et al., 2022). Tidal marshes are often restored in areas with high salinity (>15 ppt), and can be colonized by a range of plant species adapted to those conditions (Craft, 2007; Negandhi et al., 2019). Several techniques are used to restore tidal marshes (Billah et al., 2022; Craft, 2022; Mason et al., 2023) that can enhance carbon sequestration (Macreadie et al., 2017; Kelleway et al., 2020). Common techniques include the placement of dredge material on subsiding marshes or construction of new marshes by filling open water areas (Costa-Pierce and Weinstein, 2002; Madrid et al., 2012). Hydrology, including salinity, tidal flow and water level conditions, are major drivers influencing tidal marsh productivity and the success of restoration. Hydrological restoration involves diverting river water (Baustian et al., 2023) and de-embankment of existing dikes or enlarging culverts (Wolters et al., 2005; Karberg et al., 2018). Tidal reinstatement is used for restoring marshes on marginal agricultural lands in coastal areas (Kelleway et al., 2020; Lovelock et al., 2022), reintroducing tidal connections through the installation, removal or modification of water regulation structures.

The time needed for tidal marsh restoration to achieve ecological equivalence to natural marshes can vary between 3 and >15 years (Broome et al., 2019; Billah et al., 2022), but significant increases in carbon stocks (up to 3 times; Shao et al., 2022) can be reached as early as 4 years (O’Connor et al., 2020). Annual aboveground carbon production was 200–1,700 g C m^−2^ in constructed wetlands of the United States (from placement of dredged material) after 2–3 years (Madrid et al., 2012). Restored wetlands in China also had aboveground biomass stocks near 167 g C m^−2^, which were three times higher than degraded wetlands (Shao et al., 2022). The total carbon soil stock (to 1 m) of restored marshes can be about 51.86 Mg C ha^−1^, approximately two times higher than that of degraded wetlands (Shao et al., 2022).

Annual fluxes are often measured and combined to assess net carbon (or CO_2_ equivalence) benefits (Baustian et al., 2023). Aboveground and belowground net primary production rates of restored marshes in the United States can increase by four times (depending on sediment treatment) compared to degraded sites (Stagg and Mendelssohn, 2010). Soil carbon accumulation rates vary in restored tidal marshes in comparison to natural marshes, with some studies indicating the rates are lower than those of natural marshes (Broome et al., 2019, Kelsall et al., 2023, Table 1), whereas other studies indicated soil carbon accumulation rates of restored salt marshes can be nearly twice as high as reference marshes (Poppe and Rybczyk, 2021; Mason et al., 2023). The direction of this relationship may be somewhat independent of restoration, and more influenced by biophysical processes and plant species composition (Mason et al., 2023).

**Table 1.** Indicative carbon abatement benefits (g C m^−2^ yr^−1^) from soil carbon accumulation rates (from 10 to 30 cm depths) and greenhouse gas fluxes of tidal marsh restoration (see references for details on marsh ages, geomorphic settings etc.)

	Tidal Marsh		
Carbon flux	References (location)	Restored	Natural
Soil carbon accumulation (g C m^−2^ yr^−1^)	−42 to −125	−91 to −329	Craft et al. (1999) (USA), Cammen (1975) (USA), Hansen and Nestlerode (2014) (USA), Baustian et al. (2017), Baustian et al. (2020) (USA)
CO_2_ flux (g C m^−2^ yr^−1^)	+345 to +2,907	+664 to +2,141	Poffenbarger et al. (2011) (USA), Burden et al. (2013) (UK), Negandhi et al. (2019) (Australia), Shiau et al. (2019) (USA), Iram et al. (2022) (Australia)
CH_4_ flux (g C m^−2^ yr^−1^	+0.03 to +1.5	−1.2 to +0.2	Poffenbarger et al. (2011) (USA), Adams et al. (2012) (UK), Burden et al. (2013) (UK), Kroeger et al. (2017) (global), Negandhi et al. (2019) (Australia), Shiau et al. (2019) (USA), Al‐Haj and Fulweiler (2020) (global), Iram et al. (2022) (Australia)

*Note*: For a global review of carbon fluxes of restored salt marshes, see also Mason et al. (2023).

Anoxic soil conditions promote greenhouse gas emissions, reducing the magnitude of carbon abatement benefits (Emery and Fulweiler, 2017). Emissions of CO_2_ or CH_4_ from restored tidal marshes are highly variable and influenced by the legacy of restored soil (Iram et al., 2022), hydrology, vegetation (Derby et al., 2022) and elevation levels (Negandhi et al., 2019). Emissions are also strongly influenced by salinity regime (Poffenbarger et al., 2011; Kroeger et al., 2017). Restored tidal marshes are considered most effective in providing carbon abatement because microbial communities are influenced by salinity and tidal exchange, resulting in reduced greenhouse gas fluxes (Kroeger et al., 2017; Negandhi et al., 2019). Restored tidal marshes had CO_2_ and CH_4_ fluxes (Adams et al., 2012; Burden et al., 2013; Negandhi et al., 2019; Iram et al., 2022; Table 1) within the range of reference marshes and global averages for natural saltmarshes (Poffenbarger et al., 2011; Kroeger et al., 2017; Rosentreter et al., 2021). However, high precipitation events cause freshening, thus buffering salinity and reducing the carbon abatement potential of restored salt marshes during such events (Negandhi et al., 2019).

### Seagrasses

Seagrass restoration has been conducted for many decades (van Katwijk et al., 2016; Ward and Beheshti, 2023). Although the scale of restoration is often small compared to other coastal ecosystems, newer techniques in seed-based restoration (Tan et al., 2020) and mimicry of emergent traits (Temmink et al., 2020) can facilitate large-scale planting and increase restoration effectiveness (van Katwijk et al., 2009). Several guidelines focus on seagrass restoration at regional (e.g., Western Indian Ocean Region) or national (e.g., Sweden, Kiribati, United Kingdom and Ireland) scales (e.g., UNEP, 2020; Gamble et al., 2021).

Although successful seagrass restorations have been documented (van Katwijk et al., 2016; Orth et al., 2020; Tan et al., 2020), few report carbon stock recovery and sequestration in restored meadows. Those that have been assessed for carbon recovery primarily occur in the temperate coastal bays of Virginia in the mid-Atlantic, United States, the subtropical/tropical Gulf of Mexico, United States and temperate southern Australia (Table 2). Measurements here show that: (i) restored meadows have comparable or higher carbon burial rates and surface sediment carbon stocks compared to mature meadows (McGlathery et al., 2012; Marbà et al., 2015; Thorhaug et al., 2017; Orth et al., 2020); (ii) estimates can be made regarding timing, trajectory, spatial patterns and sources of blue carbon recovery following seagrass restoration (McGlathery et al., 2012; Greiner et al., 2013, 2016; Oreska et al., 2017a,b) and (iii) there are impacts of disturbance and natural recovery on carbon (Marbà et al., 2015; Thorhaug et al., 2017).

**Table 2.** Summary of specific studies investigating seagrass carbon benefits through habitat restoration

Location(s)	Bioregion and climate	Species	Carbon benefits	References
Virginia Coast Reserve Long Term Ecological Research site, Virginia coastal bays, U.S.A.	North Atlantic, temperate	*Zostera marina*	Sediment carbon content doubled in top 5 cm after 9 y (bare = 138.7 g C m^−2^, restored 9 y = 278.9 g C m^−2^); Carbon storage (including sediment and vegetation) was 3× that of bare sediments after 9 y; Carbon burial rate doubled after a 5–year lag period at start of restoration; Carbon burial rate was 36.68 g C m^−2^ y^−1^ after 10 y compared to non–depositional in unvegetated;	McGlathery et al. (2012), Greiner et al. (2013, 2016), Reynolds et al. (2016), Oreska et al. (2017a,b, 2020), Orth et al. (2020), Aoki et al. (2021)
Gulf of Mexico; Biscayne Bay, Florida, and Corpus Christi Bay, Galveston Bay, and Predator Island Laguna Madre, Texas, U.S.A.	Tropical Atlantic, tropical	*Thalassia testudinum, Halodule wrightii*, *Halophila decipiens, Syringodium filiforme*	Mean sediment carbon stocks to 20 cm in restored sites that had previously been seagrass were 38.7 ± 13.1 Mg ha^−1^, comparable or higher than natural meadows; Mean carbon gains to 20 cm =20.96 Mg C_org_ ha^−1^ Carbon burial rates were higher in restored beds of intermediate–age (4–15 y) compared to young (<3 y) or old (> 15 y)	Thorhaug et al. (2017)
False Bay, South Australia	Southern Australia, temperate	*Posidonia australis*	Radionucleotide dating failed due to low depositional and high hydrodynamic environment, leading to no estimates for carbon burial rates Carbon stocks showed a general trend of being highest in intact seagrasses, followed by recovered seagrass, and non–recovered (5.7 ± 1.2, 4.5 ± 0.7, and 3.3 ± 0.3 kg C_org_ m^−2^, respectively), but were not significantly different	Lafratta et al. (2020)
Oyster Harbor, Western Australia	Southern Australia, temperate	*Posidonia australis*	Carbon burial rates increased with age and were functionally equivalent to mature meadows after 18 years (26.4 ± 0.8 g C m^−2^ y^−1^)	Marbà et al. (2015)

In Virginia, where seagrass meadows have been restored via seed broadcasting since 2001 (McGlathery et al., 2012; Orth et al., 2020), carbon stocks in the top 5 cm of sediment in the meadow were twice that of adjacent bare sediments after 9 years (278.9 compared to 138.7 g C m^−2^; McGlathery et al., 2012) and 1.3× greater than that of younger (1–5 year old) areas of the meadow (Orth et al., 2020). The carbon burial rate was significantly higher in 10-year-old meadows (36.68 g C m^−2^ y^−1^) compared to 4-year-old meadows and unvegetated areas (no net accumulation), with a 5-year lag period after seeding before carbon burial rates doubled. Sequestration rates were expected to reach functional equivalence to mature meadows by year 12 after seeding based on projections of continued increases in shoot density (Greiner et al., 2013). This observed and expected increase in carbon sequestration occurred in parallel to a fining of the sediments and increases in shoot density with age. However, 12 years after restoration, the distribution of C_org_ concentrations at the meadow scale was driven by hydrodynamics, rather than the age of the seagrass within the meadow, with higher C_org_ concentration further into the meadow away from the bare subtidal edge due to current attenuation (Oreska et al., 2017a). Stable isotope analysis indicated that the sources of sediment organic matter in the restored seagrass meadows were distinct from those of bare sediment, and were on average composed of ~40–50% from seagrass and 46–56% from benthic macroalgae and/or seston produced in situ, with only ~3–10% coming from macroalgae or adjacent *Spartina alterniflora* salt marshes in restored meadows spanning 4–13 years old (Greiner et al., 2016; Oreska et al., 2017b).

In southwest Australia, carbon burial rates in restored meadows also increased with age, and reached functional equivalence to mature meadows 18 years after planting (mean carbon burial rate 26.4 ± 0.8 g C m^−2^ y^−1^; Marbà et al., 2015). In the Gulf of Mexico, seagrass meadows restored in eight previously disturbed areas of seagrass loss had higher 20-cm-deep sediment carbon stocks (mean C_org_ stocks 38.7 ± 13.1 Mg ha^−1^) than impacted barren or always barren sediments, with the highest carbon stocks in older restored beds. These values were comparable to or higher than natural seagrass meadows, suggesting that seagrass restoration can reduce carbon losses from previous disturbances to seagrass meadows (Thorhaug et al., 2017). Mean organic carbon gains in the top 20 cm of sediment in restored sites, compared to impacted (now barren) sites, were estimated as 20.96 Mg C_org_ ha^−1^; however, carbon accumulation rates varied significantly by site and restoration age (but not species), with intermediate-aged seagrasses (4–15 years old) having the highest carbon accumulation rates compared to young (<3 years old) or old (>15 years old) restored seagrasses (Thorhaug et al., 2017).

A complete understanding of the greenhouse gas offset potential of seagrass restoration requires a full inventory of carbon fluxes. The first complete inventory for a restored meadow (in Virginia) estimated net offsets of 0.42 t CO_2_e ha^−1^ yr.^−1^, for which the financial benefit based on carbon sequestration would only cover about 10% of restoration costs, suggesting additional ecosystem services should be assessed for incentivization (Oreska et al., 2020). Recent multidisciplinary modeling approaches have combined extensive knowledge of species’ growth and the C_org_ benefits through restoration (Duarte et al., 2013; Reynolds et al., 2016), which can demonstrate the long-term potential of seagrass restoration to capture and store carbon.

One challenge in understanding carbon dynamics following seagrass restoration is geographical biases in data collection and availability, with restoration projects mainly documented in the temperate and subtropical coastlines of North America, Europe, East Asia and Australia (van Katwijk et al., 2016), and blue carbon benefits only recorded in studies in the United States and Australia (Table 1). This is despite key academic and management interest in restoring tropical seagrasses for climate change mitigation (Rifai et al., 2023). Similarly, most studies focused on the restoration of temperate species, with a single study assessing C_org_ benefits of restoration of tropical species (Table 2). In tropical regions, several seagrass species co-occur within one meadow, adding further complexity to restoration. The traits of tropical species are highly variable, from fast-growing colonizing species to long-lived persistent species, with different responses to disturbance (Kilminster et al., 2015). Multispecies restoration, such as a combination of the five species that include all species traits (colonizing, opportunistic and persistent), has been shown to have the highest restoration potential in Indonesia (Williams et al., 2017; Asriani et al., 2018), and the combined restoration of colonizing and persistent species resulted in high organic carbon stocks in the Gulf of Mexico across 15–16 years and 38–43 years since restoration (Thorhaug et al., 2017).

While seagrass restoration can restore carbon sequestration processes (Table 2), many studies have not demonstrated additionality – the additional carbon generated as a result of an intended management action, which is a prerequisite for the calculation of carbon credits. Most restoration projects measure success based on metrics related to habitat attributes (e.g., shoot density) rather than the return of ecosystem services (Orth et al., 2020). For studies that do measure changes in carbon, some approaches (such as radionuclides to determine soil carbon accumulation rates) require years of sediment accumulation, and natural seagrass sediment dynamics such as resuspension and mixing can prevent the estimation of sediment and carbon accumulation rates (Lafratta et al., 2020). Other methods of measuring sediment accumulation to quantify carbon sequestration in seagrass meadows have proven difficult, such as modifications to the Rod Surface Elevation Table method to apply it to seagrass meadows, due to problems with measuring surface elevation change below the water surface, and maintaining feldspar marker horizons (Potouroglou et al., 2017; but see Ewers Lewis and McGlathery, 2023). Quantification of changes in carbon dynamics is important to measure because not all restored meadows accumulate substantial volumes of additional carbon. Meadows exposed to high hydrodynamic energy may lack sediment accumulation, which can cause sediment mixing and erosion to be present decades after restoration/recovery (Lafratta et al., 2020). Finally, uncertainties surrounding the influence of restoration on emissions of CH_4_, N_2_O and CO_2_, represent a global knowledge gap (Oreska et al., 2020), and accounting for emissions may result in low carbon benefits for some seagrass restoration projects.

## Challenges to successful restoration

**Figure 2.**
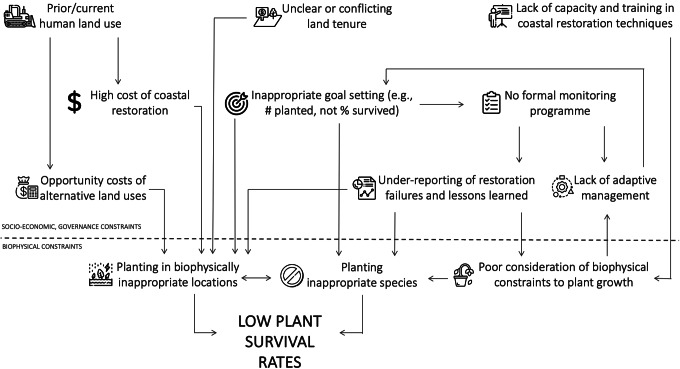
Interlinked socioeconomic, governance and biophysical constraints can lead to low blue carbon restoration success.

Regardless of restoration technique, blue carbon benefits will only be realized if restoration is conducted successfully. All restoration techniques ultimately require correct biophysical conditions within the site to allow successful planted or natural seedling establishment. While data availability on restoration outcomes is generally poor (Eger et al., 2022; Gatt et al., 2022), the global track record in restoring blue carbon ecosystems is considered to be mixed. For example, most mangrove projects in Sri Lanka showed 0% survival, with only three sites >50% (Kodikara et al., 2017). Similarly, in Colombia, only 24% of the projects were highly successful (Rodríguez-Rodríguez et al., 2021). For seagrass projects along the United States west coast, ~60% were unsuccessful (Ward and Beheshti, 2023). Restoration, whether through planting or the encouragement of natural regeneration, is challenging because seedling mortality is generally high even in natural intertidal systems (e.g., van Regteren et al., 2020; Sloey et al., 2022), and seedlings are often not planted in biophysically suitable conditions. Restoration attempts in suboptimal locations occur primarily due to socioeconomic and governance reasons (Friess et al., 2022b; Figure 2). This could include conflicting land tenure claims in suitable areas, pushing restoration activities into lower intertidal areas with fewer tenure conflicts, or ambitious short-term planting targets that require large extents of plantable areas to be found at short notice (e.g., Wodehouse and Rayment, 2019).

### Biophysical constraints to blue carbon restoration

The mortality of seedlings will be high if biophysical conditions are unsuitable for establishment. Generally, this means the physical environment of a restoration site must match the original ecohydrological conditions before disturbance. In mangroves and tidal marshes, this often relates to changes in the hydroperiod that are currently unsuitable wetland plants. For example, site elevations change substantially when aquaculture ponds are dug out, or when reclaimed areas are disconnected from further sediment input compared to the surrounding intertidal area. Other site conditions constraining wetland vegetation colonization include high sediment consolidation and low microtopography (Brooks et al., 2015).

Once the physical factors constraining natural reestablishment are understood, a key management step in wetland restoration is the removal or alleviation of that biophysical stressor (Lewis, 2005). In intertidal projects, these are often site-based engineering steps, such as manipulating site elevations through sediment addition (e.g., Staver et al., 2020), and strategic dyke breaching (e.g., Kiesel et al., 2020; López‐Portillo et al., 2021) to allow adequate water flows. However, removing constraints to natural reestablishment can be particularly challenging in seagrass restoration, as this ecosystem is often affected by stressors that occur at a distance from the restoration site, such as eutrophication caused by organic pollution from the surrounding watershed (van Katwijk et al., 2016). Lack of freshwater input due to impoundment and manipulation of the wider watershed has also been a constraint to successful coastal wetland vegetation establishment (Liu et al., 2021).

### Socioeconomic and governance constraints to blue carbon restoration

While biophysical variables determine the successful establishment of individual plants, the reasons why restoration projects are placed in suboptimal conditions in the first place are often the consequence of broader socioeconomic and governance constraints. Specifically, current land use may prohibit restoration back to the original blue carbon ecosystem; coastlines reclaimed for urban development have substantially less restoration potential than other land uses, for example (Worthington et al., 2020).

For intertidal areas where human use has been abandoned, restoration (particularly in tropical regions) can be inhibited by unclear or conflicting land tenure. Clarifying the land tenure landscape along many coastlines involves substantial investigation and negotiation, which will take time that may not be available for a project, pushing restoration efforts to locations with fewer land tenure concerns, such as commons or state lands in the subtidal zone that are not suitable for plant growth (Lovelock and Brown, 2019; Friess et al., 2022b). Land tenure can be an obstacle to restoration even in developed bureaucracies. For example, challenges are faced when negotiating permission from private landholders, persuading risk-averse public stakeholders that restoration on their lands will be successful, or where ownership boundaries on the coast are still ambiguous (Bell-James et al., 2023).

Inappropriate goal setting can also lead to poor restoration outcomes (Waltham et al., 2021), such as planting in inappropriate locations. Many projects set the number of seedlings planted as a goal. Planting over the short time scales mandated by funders may encourage managers to seek large open areas (such as undervalued tidal flats, sensu Chen and Lee, 2022) that provide sufficient space for planting but are not biophysically suitable for vegetation establishment (Lovelock and Brown, 2019; Wodehouse and Rayment, 2019). Ultimately, coastal restoration will be most successful when targets are based on robust knowledge of past and future changes that underpin suitable scenarios of environmental performance (Sheaves et al., 2021), which allows for long-term, iterative and adaptive management (e.g., Thom, 2000).

Finally, coastal restoration costs can be high compared to terrestrial restoration, due to costs such as construction actions for hydrological manipulation (Bayraktarov et al., 2016). Carbon accounting of a rehabilitated seagrass meadow in the South Bay, Virginia, United States suggests that blue carbon credit generation may only offset ~10% of restoration costs (Oreska et al., 2020). Costs can sometimes be reduced by community labor for planting and other activities (e.g., Tan et al., 2020), although this is not often the major cost in a restoration project. As such, blue carbon projects that involve restoration are currently not breaking even, and future projects will likely require cofinancing options such as tax incentives or philanthropic funding in addition to carbon credit sales (Friess et al., 2022a). Combined, socioeconomic and governance factors have important implications for the scale of restoration; the area biophysically suitable for mangrove restoration in Southeast Asia is estimated at >303,000 ha, but only 5.5–34.2% of this area was deemed restorable once financial, land use and operational constraints were considered (Zeng et al., 2020).

### Spatial prioritization of blue carbon restoration

Many restoration projects are opportunistic in their location (e.g., Ledoux et al., 2005) due to low land availability and opportunity costs. However, achieving ambitious restoration targets and meaningful blue carbon gains requires landscape-scale planning. Modeling can consider various biophysical, socioeconomic and governance constraints when determining optimal restoration locations (e.g., Syahid et al., 2023), and has been widely used in predicting potential changes in the distribution of mangroves (Hu et al., 2020; Rodriguez-Medina et al., 2020; Sahana et al., 2022), seagrasses (Bertelli et al., 2022) and tidal marshes (Boumans et al., 2002; Raw et al., 2021). Species distribution models have been specifically used to determine suitable areas for seagrass (Valle et al., 2011; Stankovic et al., 2019) and mangrove (Hu et al., 2020) restoration. These models predict the environmental distribution of species and can be projected into geographical space as habitat suitability, probability of species occurrence or favorability of species occurrence, which can be interpreted as restoration potential (Stankovic et al., 2019). The positive correlation of the models’ habitat suitability prediction and the planting units of seagrass (Valle et al., 2015) demonstrated that usage of the model can eliminate “best professional judgment” for site selection (Stankovic et al., 2019). However, most models rely on complex relationships of the abiotic factors that shape a realized niche at a specific point in time, rather than the fundamental niche (Grech and Coles, 2010), which can provide greater potential extents and additional restoration sites, resulting in an increase in total seagrass coverage (Oreska et al., 2021). In addition, these models, coupled with spatial drivers and models of blue carbon variability (e.g., Ewers Lewis et al., 2019), offer guidelines for the return of the ecosystem services through restoration across spatial and temporal scales.

Modeling can also be used to show how the realized/fundamental niche may be modified as physical conditions change with climate change, such as increasing inundation periods under sea-level rise. This approach has most commonly been applied to conservation projects by assessing the sustainability of existing blue carbon resources and their projected distributions in the future (e.g., Rosencranz et al., 2019 for tidal marshes; Nguyen et al., 2022 for seagrasses and mangroves). Restoration suitability is implicit in these models when they predict landward migration into presently terrestrial areas, as those areas will need to be colonized by new intertidal vegetation. While less common, intertidal habitat restoration has also been specifically modeled under sea-level rise, with biogeomorphic models of a planned tidal marsh restoration project showing that newly colonizing vegetation will be able to keep pace with realistic rates of projected sea-level rise, and resilience is particularly influenced by restoration method (Gourgue et al., 2022).

## Conclusions

Extensive habitat loss creates ample opportunity for the restoration of mangroves, tidal marshes and seagrasses. The evidence is clear that their restoration leads to substantial per hectare gains in blue carbon, and has led to rapid interest in blue carbon restoration for the achievement of climate change mitigation policy. While the realizable area of restoration globally is much smaller than its biophysical potential, future blue carbon restoration is likely to make a meaningful contribution to climate change mitigation, while providing a wide range of co-benefits to coastal communities around the world.

The important carbon gains and other co-benefits from blue carbon restoration will only be unlocked, however, if success rates and scales of restoration increase substantially beyond what is currently being achieved around the world. Restoration projects, particularly for mangroves and seagrasses, often struggle to meet their success criteria due to myriad biophysical, socioeconomic and governance constraints. Issues of land tenure and opportunity costs often push mangrove restoration projects into uncontested but suboptimal spaces in the lower intertidal or upper subtidal zone, or seagrasses are restored in areas where the main driver of ecosystem degradation (such as water pollution) has not been addressed. However, guidelines and examples exist to overcome such barriers, and improve the success of coastal habitat restoration and the carbon benefits they provide.

## Data Availability

No primary data were generated for this manuscript.

## References

[r1] Adame MF, Santini NS, Torres-Talamante O and Rogers K (2021) Mangrove sinkholes (cenotes) of the Yucatan peninsula, a global hotspot of carbon sequestration. Biology Letters 17, 20210037. 33947219 10.1098/rsbl.2021.0037 PMC8097219

[r2] Adams CA, Andrews JE and Jickells T (2012) Nitrous oxide and methane fluxes vs carbon, nitrogen and phosphorous burial in new intertidal and saltmarsh sediments. Science of the Total Environment 434, 240–251. 22197113 10.1016/j.scitotenv.2011.11.058

[r3] Al‐Haj AN and Fulweiler RW (2020) A synthesis of methane emissions from shallow vegetated coastal ecosystems. Global Change Biology 26, 2988–3005. 32068924 10.1111/gcb.15046

[r4] Andreetta A, Huertas AD, Lotti M and Cerise S (2016) Land use changes affecting soil organic carbon storage along a mangrove swamp rice chronosequence in the Cacheu and Oio regions (northern Guinea-Bissau). Agriculture, Ecosystems & Environment 216, 314–321.

[r5] Aoki LR, McGlathery KJ, Wiberg PL, Oreska MP, Berger AC, Berg P and Orth RJ (2021) Seagrass recovery following marine heat wave influences sediment carbon stocks. Frontiers in Marine Science 7, 576784.

[r6] Asriani N, Ambo-Rappe R, Lanuru M and Williams SL (2018) Species richness effects on the vegetative expansion of transplanted seagrass in Indonesia. Botanica Marina 61, 205–211.

[r7] Azman MS, Sharma S, Liyana Hamzah M, Mohamad Zakaria R, Palaniveloo K and MacKenzie RA (2023) Total ecosystem blue carbon stocks and sequestration potential along a naturally regenerated mangrove forest chronosequence. Forest Ecology and Management 527, 120611.

[r8] Baustian MM, Jung H, Bienn HC, Barra M, Hemmerling SA, Wang Y, White E and Meselhe E (2020) Engaging coastal community members about natural and nature-based solutions to assess their ecosystem function. Ecological Engineering 143, 100015.

[r9] Baustian MM, Liu B, Moss L, Dausman A and Pahl JW (2023) Climate change mitigation potential of Louisiana’s coastal area: Current estimates and future projections. Ecological Applications 33, e2847. 36932861 10.1002/eap.2847

[r10] Baustian MM, Stagg CL, Perry CL, Moss LC, Carruthers TJB and Allison M (2017) Relationships between salinity and short-term soil carbon accumulation rates from marsh types across a landscape in the Mississippi River Delta. Wetlands 37, 313–324.

[r11] Bayraktarov E, Saunders MI, Abdullah S, Mills M, Beher J, Possingham HP, Mumby PJ and Lovelock CE (2016) The cost and feasibility of marine coastal restoration. Ecological Applications 26, 1055–1074. 27509748 10.1890/15-1077

[r12] Bell-James J, Fitzsimons JA and Lovelock CE (2023) Land tenure, ownership and use as barriers to coastal wetland restoration projects in Australia: Recommendations and solutions. Environmental Management 72, 179–189. 37010555 10.1007/s00267-023-01817-w PMC10220139

[r13] Bertelli CM, Stokes HJ, Bull JC and Unsworth RKF (2022) The use of habitat suitability modelling for seagrass: A review. Frontiers in Marine Science 9, 997831.

[r14] Billah MM, Bhuiyan MKA, Islam MA, Das J and Hoque AR (2022) Salt marsh restoration: An overview of techniques and success indicators. Environmental Science and Pollution Research 29, 15347–15363. 34989993 10.1007/s11356-021-18305-5

[r15] Boumans RMJ, Burdick DM and Dionne M (2002) Modeling habitat change in salt marshes after tidal restoration. Restoration Ecology 10, 543–555.

[r16] Bourderesque C-F, Blanfuné A, Pergent G and Thibaut T (2021) Restoration of seagrass meadows in the Mediterranean Sea: A critical review of effectiveness and ethical issues. Water 13, 1034.

[r17] Brooks KL, Mossman HL, Chitty JL and Grant A (2015) Limited vegetation development on a created salt marsh associated with over-consolidated sediments and lack of topographic heterogeneity. Estuaries and Coasts 38, 325–336.

[r18] Broome SW, Craft CB and Burchell MR (2019) Tidal marsh creation. In Coastal Wetlands. Amsterdam: Elsevier, pp. 789–816.

[r19] Burden A, Garbutt RA, Evans CD, Jones DL and Cooper DM (2013) Carbon sequestration and biogeochemical cycling in a saltmarsh subject to coastal managed realignment. Estuarine, Coastal and Shelf Science 120, 12–20.

[r20] Cameron C, Hutley LB, Friess DA and Brown B (2019) High greenhouse gas emissions mitigation benefits from mangrove rehabilitation in Sulawesi, Indonesia. Ecosystem Services 40, 101035.

[r21] Cammen LM (1975) Accumulation rate and turnover time of organic carbon in a salt marsh sediment. Limnology and Oceanography 20, 1012–1015.

[r22] Campbell AD, Fatoyinbo L, Goldberg L and Lagomasino D (2022) Global hotspots of salt marsh change and carbon emissions. Nature 612, 701–706. 36450979 10.1038/s41586-022-05355-z PMC9771810

[r23] Carnell PE, Palacios MM, Waryszak P, Trevathan-Tackett SM, Masqué P and Macreadie PI (2022) Blue carbon drawdown by restored mangrove forests improves with age. Journal of Environmental Management 306, 114301. 35032938 10.1016/j.jenvman.2021.114301

[r24] Chen ZL and Lee SY (2022) Tidal flats as a significant carbon reservoir in global coastal ecosystems. Frontiers in Marine Science 9, 900896.

[r25] Costa-Pierce BA and Weinstein MP (2002) Use of dredge materials for coastal restoration. Ecological Engineering 3, 181–186.

[r26] Cotavicz LC Jr , Abril G, Sanders CJ, Tait DR, Sippo JZ et al. (2024) Methane oxidation minimizes emissions and offsets to carbon burial in mangroves. Nature Climate Change. 14, 275–281.

[r27] Craft C (2007) Freshwater input structures soil properties, vertical accretion, and nutrient accumulation of Georgia and U.S. tidal marshes. Limnology and Oceanography 53, 1220–1230.

[r28] Craft C (2022) Creating and Restoring Wetlands: From Theory to Practice, 2nd Edn. Amsterdam: Elsevier.

[r29] Craft C, Reader J, Sacco JN and Broome SW (1999) Twenty-five years of ecosystem development of constructed Spartina Alterniflora (loisel) marshes. Ecological Applications 9, 1405–1419.

[r30] Derby RK, Needelman BA, Roden AA and Megonigal JP (2022) Vegetation and hydrology stratification as proxies to estimate methane emission from tidal marshes. Biogeochemistry 157, 227–243.

[r31] Duarte CM, Sintes T and Marbà N (2013) Assessing the CO2 capture potential of seagrass restoration projects. Journal of Applied Ecology 5, 1341–1349.

[r32] Dung LV, Tue NT, Nhuan MT and Omori K (2016) Carbon storage in a restored mangrove forest in Can Gio mangrove Forest Park, Mekong Delta. Vietnam. Forest Ecology and Management 380, 31–40.

[r33] Dunic JC, Brown CJ, Connolly RM, Turschwell MP and Côté IM (2021) Long‐term declines and recovery of meadow area across the world’s seagrass bioregions. Global Change Biology 27, 4096–4109. 33993580 10.1111/gcb.15684

[r34] Eger AM, Earp HS, Friedman K, Gatt Y, Hagger V, Hancock B, Kaewsrikhaw R, Mcleod E, Moore AM and Niner HJ (2022) The need, opportunities, and challenges for creating a standardized framework for marine restoration monitoring and reporting. Biological Conservation 266, 109429.

[r35] Ellison AM, Felson AJ and Friess DA (2020) Mangrove rehabilitation and restoration as experimental adaptive management. Frontiers in Marine Science 7, 327.

[r36] Emery HE and Fulweiler RW (2017) Incomplete tidal restoration may lead to persistent high CH4 emission. Ecosphere 8, e01968.

[r37] Ewers Lewis CJ and McGlathery KJ (2023) A novel subsurface sediment plate method for quantifying sediment accumulation and erosion in seagrass meadows. Frontiers in Marine Science 10, 1232619.

[r38] Ewers Lewis CJ, Young MA, Ierodiaconou D, Baldock JA, Hawke B, Sanderman J, Carnell PE and Macreadie PI (2019) Drivers and modelling of blue carbon stock variability. Biogeosciences 17, 2041–2059.

[r39] Fluet-Chouinard E, Stocker BD, Zhang Z, Malhotra A, Melton JR, Poulter B, Kaplan JO, Goldewijk KK, Siebert S and Minayeva T (2023) Extensive global wetland loss over the past three centuries. Nature 614, 281–286. 36755174 10.1038/s41586-022-05572-6

[r40] Fourqurean JW, Duarte CM, Kennedy H, Marbà N, Holmer M, Mateo MA, Apostolake ET, Kendrick GA, Krause-Jensen D, McGlathery KJ and Serrano O. (2012) Seagrass ecosystems as a globally significant carbon stock. Nature Geoscience 5, 505–509.

[r41] Friess DA (2023) The potential of mangrove and seagrass blue carbon for Small Island states. Current Opinion in Environmental Sustainability 64, 101324.

[r42] Friess DA, Gatt YM, Ahmad R, Brown BM, Sidik F and Wodehouse D (2022b) Achieving ambitious mangrove restoration targets will need a transdisciplinary and evidence-informed approach. One Earth 5, 456–460.

[r43] Friess DA, Howard J, Huxham M, Macreadie PI and Ross F (2022a) Capitalizing on the global financial interest in blue carbon. PLOS Climate 1, e0000061.

[r44] Friess DA, Rogers K, Lovelock CE, Krauss KW, Hamilton SE, Lee SY, Lucas R, Primavera J, Rajkaran A and Shi S (2019) The state of the world’s mangrove forests: Past, present, and future. Annual Review of Environment and Resources 44, 89–115.

[r45] Gamble C, Debney A, Glover A, Bertelli C, Green B et al. (2021) Seagrass Restoration Handbook UK & Ireland. London, UK: Zoological Society of London.

[r46] Garbutt A and Wolters M (2008) The natural regeneration of salt marsh on formerly reclaimed land. Applied Vegetation Science 11, 335–344.

[r47] Gatt YM, Andradi-Brown DA, Ahmadia GN, Martin PA, Sutherland WJ, Spalding MD, Donnison A and Worthington TA (2022) Quantifying the reporting, coverage and consistency of key indicators in mangrove restoration projects. Frontiers in Forests and Global Change 5, 720394.

[r48] Gourgue O, van Belzen J, Schwarz C, Vandenbruwaene W, Vanlede J, et al. (2022) Biogeomorphic modeling to assess the resilience of tidal-marsh restoration to sea level rise and sediment supply. Earth Surface Dynamics 10, 431–553.

[r49] Grech A and Coles RG (2010) An ecosystem-scale predictive model of coastal seagrass distribution. Aquatic Conservation: Marine and Freshwater Ecosystems 20, 437–444.

[r50] Greiner JT, McGlathery KJ, Gunnell J and McKee BA (2013) Seagrass restoration enhances “blue carbon” sequestration in coastal waters. PLoS One 8, e72469. 23967303 10.1371/journal.pone.0072469 PMC3743776

[r51] Greiner JT, Wilkinson GM, McGlathery KJ and Emery KA (2016) Sources of sediment carbon sequestered in restored seagrass meadows. Marine Ecology Progress Series 551, 95–105.

[r52] Griscom BW, Adams J, Ellis PW, Houghton RA, Lomax G, Miteva DA, Schlesinger WH, Shoch D, Siikamäki JV, Smith P, Woodbury P, Zganjar C, Blackman A, Campari J, Conant RT, Delgado C, Elias P, Gopalakrishna T, Hamsik MR, Herrero M, Kiesecker J, Landis E, Laestadius L, Leavitt SM, Minnemeyer S, Polasky S, Potapov P, Putz FE, Sanderman J, Silvius M, Wollenberg E and Fargione J (2017) Natural climate solutions. Proceedings of the National Academy of Sciences 114, 11645–11650. 10.1073/pnas.1710465114 PMC5676916 29078344

[r53] Hagger V, Waltham NJ and Lovelock CE (2022) Opportunities for coastal wetland restoration for blue carbon with co-benefits for biodiversity, coastal fisheries, and water quality. Ecosystem Services 55, 101423.

[r54] Hamilton SE and Friess DA (2018) Global carbon stocks and potential emissions due to mangrove deforestation from 2000 to 2012. Nature Climate Change 8, 240–244.

[r55] Hansen VD and Nestlerode JA (2014) Carbon sequestration in wetland soils of the northern Gulf of Mexico coastal region. Wetlands Ecology and Management 22, 289–303.

[r56] Hu W, Wang Y, Zhang D, Yu W, Chen G, Xie T, Liu Z, Ma Z, Du J, Chao B, Lei G and Chen B (2020) Mapping the potential of mangrove forest restoration based on species distribution models: A case study in China. Science of the Total Environment 748, 142321. 33113686 10.1016/j.scitotenv.2020.142321

[r57] Iram N, Maher DT, Lovelock CE, Baker T, Cadier C and Adame MF (2022) Climate change mitigation and improvement of water quality from the restoration of a subtropical coastal wetland. Ecological Applications 32, e2620. 35389535 10.1002/eap.2620 PMC9285723

[r58] Jayathilake DR and Costello MJ (2018) A modelled global distribution of the seagrass biome. Biological Conservation 226, 120–126.

[r59] Jia M, Wang Z, Mao D, Ren C, Song K, Zhao C, Wang C, Xiao X and Wang Y (2023) Mapping global distribution of mangrove forests at 10-m resolution. Science Bulletin 68, 1302–1316. 10.1016/j.scib.2023.05.004 37217429

[r60] Johannessen SC (2022) How can blue carbon burial in seagrass meadows increase long-term, net sequestration of carbon? A critical review. Environmental Research Letters 17, 093004.

[r61] Karberg JM, Beattie KC, O’Dell DI and Omand KA (2018) Tidal hydrology and salinity drives salt marsh vegetation restoration and (*Phragmites australis*) control in New England. Wetlands 38, 993–1003.

[r62] Kelleway JJ, Serrano O, Baldock JA, Burgess R, Cannard T, Lavery PS, Lovelock CE, Macreadie PI, Masqué P, Newnham M and Saintilan N (2020) A national approach to greenhouse gas abatement through blue carbon management. Global Environmental Change 63, 102083.

[r63] Kelsall M, Quirk T, Wilson C and Snedden GA (2023) Sources and chemical stability of soil organic carbon in natural and created coastal marshes of Louisiana. Science of the Total Environment 867, 161415. 10.1016/j.scitotenv.2023.161415. 36621493

[r64] Kiesel J, Schuerch M, Christie EK, Möller I, Spencer T and Vafeidis AT (2020) Effective design of managed realignment schemes can reduce coastal flood risks. Estuarine, Coastal and Shelf Science 242, 106844.

[r65] Kilminster K, McMahon K, Waycott M, Kendrick GA, Scanes P, McKenzie L, O’Brien KR, Lyons M, Ferguson A, Maxwell P, Glasby T and Udy J (2015) Unravelling complexity in seagrass systems for management: Australia as a microcosm. Science of the Total Environment 534, 97–109. 25917445 10.1016/j.scitotenv.2015.04.061

[r66] Kirwan ML, Megonigal JP, Noyce GL and Smith AJ (2023) Geomorphic and ecological constraints on the coastal carbon sink. Nature Reviews Earth & Environment 4, 393–406.

[r67] Kodikara KAS, Mukherjee N, Jayatissa LP, Dahdouh‐Guebas F, Koedam N (2017) Have mangrove restoration projects worked? An in‐depth study in Sri Lanka. Restoration Ecology 25, 705–716.

[r68] Kroeger KD, Crooks S, Moseman-Valtierra S and Tang J (2017) Restoring tides to reduce methane emissions in impounded wetlands: A new and potent blue carbon climate change intervention. Scientific Reports 7, 1914. 28931842 10.1038/s41598-017-12138-4 PMC5607314

[r69] Kusumaningtyas MA, Kepel TL, Solihuddin T, Lubis AA, Putra ADP, Sugiharto U, Ati RNA, Salim HL, Mustikasari E, Heriati A, Daulat A, Sudirman N, Suryono DD and Rustam A (2022) Carbon sequestration potential in the rehabilitated mangroves in Indonesia. Ecological Research 37, 80–91.

[r70] Lafratta A, Serrano O, Masqué P, Mateo MA, Fernandes M, Gaylard S and Lavery PS (2020) Challenges to select suitable habitats and demonstrate ‘additionality’ in blue carbon projects: A seagrass case study. Ocean & Coastal Management 197, 105295–105295.

[r71] Ledoux L, Cornell S, O’Riordan T, Harvey R and Banyard L (2005) Towards sustainable flood and coastal management: Identifying drivers of, and obstacles to, managed realignment. Land Use Policy 22, 129–144.

[r72] Lewis RR (2005) Ecological engineering for successful management and restoration of mangrove forests. Ecological Engineering 24, 403–418.

[r73] Liu Z, Fagherazzi S, Li J and Cui B (2021) Mismatch between watershed effects and local efforts constrains the success of coastal salt marsh vegetation restoration. Journal of Cleaner Production 292, 126103.

[r74] López‐Portillo J, Zaldívar‐Jiménez A, Lara‐Domínguez AL, Pérez‐Ceballos R, Bravo‐Mendoza M, Álvarez NN and Aguirre‐Franco L (2021) Hydrological rehabilitation and sediment elevation as strategies to restore mangroves in terrigenous and calcareous environments in Mexico. In Krauss KW, Zhu Z, Stagg CL (eds.), Geophysical Monograph Series, 1st Edn. Hoboken: Wiley, pp. 173–190.

[r75] Lovelock CE, Adame MF, Bradley J, Dittmann S, Hagger V, Hickey SM, Hutley LB, Jones A, Kelleway JJ, Lavery PS and Macreadie PI (2022) An Australian blue carbon method to estimate climate change mitigation benefits of coastal wetland restoration. Restoration Ecology 26, e13739.

[r76] Lovelock CE and Brown BM (2019) Land tenure considerations are key to successful mangrove restoration. Nature Ecology & Evolution 3, 1135–1135. 31285575 10.1038/s41559-019-0942-y

[r77] Macreadie PI, Costa MDP, Atwood TB, Friess DA, Kelleway JJ, Kennedy H, Lovelock CE, Serrano O and Duarte CM (2021) Blue carbon as a natural climate solution. Nature Reviews Earth & Environment 2, 826–839.

[r78] Macreadie PI, Nielsen DA, Kelleway JJ, Atwood TB, Seymour JR, Petrou K, Connolly RM, Thomson ACG, Trevathan-Tackett SM and Ralph PJ (2017) Can we manage coastal ecosystems to sequester more blue carbon? Frontiers in Ecology and the Environment 15, 206–213.

[r79] Madrid EN, Quigg A and Armitage AR (2012) Marsh construction techniques influence net plant carbon capture by emergent and submerged vegetation in a brackish marsh in the northwestern Gulf of Mexico. Ecological Engineering 42, 54–63.

[r80] Marbà N, Arias-Ortiz A, Masqué P, Kendrick GA, Mazarrasa I, Bastyan GR, Garcia-Orellana J and Duarte CM (2015) Impact of seagrass loss and subsequent revegetation on carbon sequestration and stocks. Journal of Ecology 103, 296–302.

[r81] Marchand C (2017) Soil carbon stocks and burial rates along a mangrove forest chronosequence (French Guiana). Forest Ecology and Management 384, 92–99.

[r82] Mason VG, Burden A, Epstein G, Jupe LL, Wood KA and Skov MW (2023) Blue carbon benefits from global saltmarsh restoration. Global Change Biology 29, 6517–6545. 37746862 10.1111/gcb.16943

[r83] Matsui N, Suekuni J, Nogami M, Havanond S and Salikul P (2010) Mangrove rehabilitation dynamics and soil organic carbon changes as a result of full hydraulic restoration and re-grading of a previously intensively managed shrimp pond. Wetlands Ecology and Management 18, 233–242.

[r84] McGlathery KJ, Reynolds LK, Cole LW, Orth RJ, Marion SR and Schwarzschild A (2012) Recovery trajectories during state change from bare sediment to eelgrass dominance. Marine Ecology Progress Series 448, 209–221.

[r85] McKenzie LJ, Nordlund LM, Jones BL, Cullen-Unsworth LC, Roelfsema C and Unsworth RK (2020) The global distribution of seagrass meadows. Environmental Research Letters 15, 074041.

[r86] Negandhi K, Edwards G, Kelleway JJ, Howard D, Safari D and Saintilan N (2019) Blue carbon potential of coastal wetland restoration varies with inundation and rainfall. Scientific Reports 9, 4368. 30867475 10.1038/s41598-019-40763-8 PMC6416304

[r87] Nguyen NTH, Friess DA, Todd PA, Mazor T, Lovelock CE, et al. (2022) Maximising resilience to sea-level rise in urban coastal ecosystems through systematic conservation planning. Landscape and Urban Planning 221, 104374.

[r88] O’Connor JJ, Fest BJ, Sievers M and Swearer SE (2020) Impacts of land management practices on blue carbon stocks and greenhouse gas fluxes in coastal ecosystems—A meta-analysis. Global Change Biology 26, 1354–1366. 31799721 10.1111/gcb.14946

[r89] Oreska MPJ, McGlathery KJ, Aoki LR, Berger AC, Berg P and Mullins L (2020) The greenhouse gas offset potential from seagrass restoration. Scientific Reports 10, 7325. 32355280 10.1038/s41598-020-64094-1 PMC7193639

[r90] Oreska MPJ, McGlathery KJ and Porter JH (2017a) Seagrass blue carbon spatial patterns at the meadow-scale. PLoS One 12, 176630. 10.1371/journal.pone.0176630 PMC5407773 28448617

[r91] Oreska MPJ, McGlathery KJ, Wiberg PL, Orth RJ and Wilcox DJ (2021) Defining the *Zostera marina* (eelgrass) niche from long-term success of restored and naturally colonized meadows: Implications for seagrass restoration. Estuaries and Coasts 44, 396–411.

[r92] Oreska MPJ, Wilkinson GM, McGlathery KJ, Bost M and McKee BA (2017b) Non-seagrass carbon contributions to seagrass sediment blue carbon. Limnology and Oceanography 63, 3–18.

[r93] Orth RJ, Lefcheck JS, McGlathery KJ, Aoki LR, Luckenbach MW, Moore KA, Oreska MPJ, Snyder R, Wilcox DJ and Lusk B (2020) Restoration of seagrass habitat leads to rapid recovery of coastal ecosystem services. Science Advances 6, 6434. 10.1126/sciadv.abc6434 PMC7541073 33028530

[r94] Osland MJ, Spivak AC, Nestlerode JA, Lessmann JM, Almario AE, Heitmuller PT, Russell MJ, Krauss KW, Alvarez F, Dantin DD, Harvey JE, From AS, Cormier N and Stagg CL (2012) Ecosystem development after mangrove wetland creation: Plant–soil change across a 20-year chronosequence. Ecosystems 15, 848–866.

[r95] Pham VH, Luu VD, Nguyen TT and Koji O (2017) Will restored mangrove forests enhance sediment organic carbon and ecosystem carbon storage? Regional Studies in Marine Science 14, 43–52.

[r96] Poffenbarger HJ, Needelman BA and Megonigal JP (2011) Salinity influence on methane emissions from tidal marshes. Wetlands 31, 831–842.

[r97] Poppe KL and Rybczyk JM (2021) Tidal marsh restoration enhances sediment accretion and carbon accumulation in the Stillaguamish River estuary, Washington. PLoS One 16, e0257244. 34506575 10.1371/journal.pone.0257244 PMC8432862

[r98] Potouroglou M, Bull JC, Krauss KW, Kennedy HA, Fusi M, Daffonchio D, Mangora MM, Githaiga MN, Diele K and Huxham M (2017) Measuring the role of seagrasses in regulating sediment surface elevation. Scientific Reports 7, 11917. 28928433 10.1038/s41598-017-12354-y PMC5605501

[r99] Ratul SB, Gu X, Qiao P, Sagala FW, Nan S, Islam N and Chen L (2022) Blue carbon sequestration following mangrove restoration: Evidence from a carbon neutral case in China. Ecosystem Health and Sustainability 8, 2101547.

[r100] Raw JL, Adams JB, Bornman TG, Riddin T and Vanderklift MA (2021) Vulnerability to sea-level rise and the potential for restoration to enhance blue carbon storage in salt marshes of an urban estuary. Estuarine, Coastal and Shelf Science 260, 107495.

[r101] Reynolds LK, Waycott M, McGlathery KJ and Orth RJ (2016) Ecosystem services returned through seagrass restoration. Restoration Ecology 24, 583–588.

[r102] Rifai H, Quevedo JMD, Lukman KM, CFA S, Risandi J, Hernawan UE, Uchiyama Y, Ambo-Rappe R and Kohsaka R (2023) Potential of seagrass habitat restorations as nature-based solutions: Practical and scientific implications in Indonesia. Ambio 52, 546–555. 36484926 10.1007/s13280-022-01811-2 PMC9849659

[r103] Rivera-Monroy VH, Zhao X, Wang H and Xue ZG (2022) Are existing modeling tools useful to evaluate outcomes in mangrove restoration and rehabilitation projects? A mini review. Forests 13, 1638.

[r104] Rodriguez-Medina K, Yanez-Arenas C, Peterson AT, Euan Avila J and Herrera-Silveira J (2020) Evaluating the capacity of species distribution modeling to predict the geographic distribution of the mangrove community in Mexico. PLoS One 15, e0237701. 32817628 10.1371/journal.pone.0237701 PMC7446832

[r105] Rodríguez-Rodríguez JA, Mancera-Pineda JE and Tavera H (2021) Mangrove restoration in Colombia: Trends and lessons learned. Forest Ecology and Management 496, 119414.

[r106] Rosencranz JA, Thorne KM, Buffington KJ, Overton CT, Takekawa JY, et al. (2019) Rising tides: Assessing habitat vulnerability for an endangered salt marsh-dependent species with sea-level rise. Wetlands 39, 1203–1218.

[r107] Rosentreter JA, Al-Haj AN, Fulweiler RW and Williamson P (2021) Methane and nitrous oxide emissions complicate coastal blue carbon assessments. Global Biogeochemical Cycles 35, e2020GB006858.

[r108] Sahana M, Areendran G and Sajjad H (2022) Assessment of suitable habitat of mangrove species for prioritizing restoration in coastal ecosystem of Sundarban biosphere reserve, India. Scientific Reports 12, 20997. 36470951 10.1038/s41598-022-24953-5 PMC9723184

[r109] Sasmito SD, Basyuni M, Kridalaksana A, Saragi-Sasmito MF, Lovelock CE and Murdiyarso D (2023) Challenges and opportunities for achieving sustainable development goals through restoration of Indonesia’s mangroves. Nature Ecology & Evolution 7, 62–70. 36593293 10.1038/s41559-022-01926-5

[r110] Sasmito SD, Taillardat P, Clendenning JN, Cameron C, Friess DA, Murdiyarso D and Hutley LB (2019) Effect of land-use and land-cover change on mangrove blue carbon: A systematic review. Global Change Biology 25, 4291–4302. 31456276 10.1111/gcb.14774

[r111] Shao P, Han H, Yang H, Li T, Zhang D, Ma J, Duan D and Sun J (2022) Responses of above-and belowground carbon stocks to degraded and recovering wetlands in the Yellow River Delta. Frontiers in Ecology and Evolution 10, 856479.

[r112] Sheaves M, Waltham NJ, Benham C, Bradley M, Mattone C, Diedrich A, Sheaves J, Sheaves A, Hernandez S and Dale P (2021) Restoration of marine ecosystems: Understanding possible futures for optimal outcomes. Science of the Total Environment 796, 148845. 34274664 10.1016/j.scitotenv.2021.148845

[r113] Shiau YJ, Burchell MR, Krauss KW, Broome SW and Birgand F (2019) Carbon storage potential in a recently created brackish marsh in eastern North Carolina, USA. Ecological Engineering 127, 579–588.

[r114] Sidik F, Lawrence A, Wagey T, Zamzani F and Lovelock CE (2023) Blue carbon: A new paradigm of mangrove conservation and management in Indonesia. Marine Policy 147, 105388.

[r115] Sloey TM, Lim KE, Moore J, Heng JM, Heng JM and Van Breugel M (2022) Influence of abiotic drivers on 1‐year seedling survival of six mangrove species in Southeast Asia. Restoration Ecology 30, e13694.

[r116] Song S, Ding Y, Li W, Meng Y, Zhou J, Gou R, Zhang C, Ye S, Saintilan N, Krauss KW, Crooks S, Lv S and Lin G (2023) Mangrove reforestation provides greater blue carbon benefit than afforestation for mitigating global climate change. Nature Communications 14, 756. 10.1038/s41467-023-36477-1 PMC9918466 36765059

[r117] Sparks EL, Cebrian J, Biber PD, Sheehan KL and Tobias CR (2013) Cost-effectiveness of two small-scale salt marsh restoration designs. Ecological Engineering 53, 250–256.

[r118] Stagg CL and Mendelssohn IA (2010) Restoring ecological function to a submerged salt marsh. Restoration Ecology 18, 10–17.

[r119] Stankovic M, Kaewsrikhaw R, Rattanachot E and Prathep A (2019) Modeling of suitable habitat for small-scale seagrass restoration in tropical ecosystems. Estuarine, Coastal and Shelf Science 231, 106465.

[r120] Staver LW, Stevenson JC, Cornwell JC, Nidzieko NJ, Staver KW, Owens MS, Logan L, Kim C and Malkin SY (2020) Tidal marsh restoration at Poplar Island: II. Elevation trends, vegetation development, and carbon dynamics. Wetlands 40, 1687–1701.

[r121] Syahid LN, Sakti AD, Ward R, Rosleine D, Windupranata W and Wikantika K (2023) Optimizing the spatial distribution of Southeast Asia mangrove restoration based on zonation, species and carbon projection schemes. Estuarine, Coastal and Shelf Science 293, 108477.

[r122] Taillardat P, Thompson BS, Garneau TK and Friess DA (2020) Climate change mitigation potential of wetlands and the cost-effectiveness of their restoration. Interface Focus 10, 20190129. 32832065 10.1098/rsfs.2019.0129 PMC7435041

[r123] Tan YM, Dalby O, Kendrick GA, Statton J, Sinclair EA, Fraser MW, Macreadie PI, Gillies CL, Coleman RA and Waycott M (2020) Seagrass restoration is possible: Insights and lessons from Australia and New Zealand. Frontiers in Marine Science 7, 617.

[r124] Temmink RJ, Christianen MJ, Fivash GS, Angelini C, Boström C, Didderen K, Engel SM, Esteban N, Gaeckle JL and Gagnon K (2020) Mimicry of emergent traits amplifies coastal restoration success. Nature Communications 11, 3668. 10.1038/s41467-020-17438-4 PMC7376209 32699271

[r125] Thom RM (2000) Adaptive management of coastal ecosystem restoration projects. Ecological Engineering 15, 365–372.

[r126] Thorhaug A, Poulos HM, López-Portillo J, Ku TCW and Berlyn, GP (2017) Seagrass blue carbon dynamics in the Gulf of Mexico: Stocks, losses from anthropogenic disturbance, and gains through seagrass restoration. Science of the Total Environment 605**–606**, 626–636. 28672251 10.1016/j.scitotenv.2017.06.189

[r127] Thura K, Serrano O, Gu J, Fang Y, Htwe HZ, Zhu Y, Huang R, Agusti S, Duarte CM, Wang H and Wu J (2023) Mangrove restoration built soil organic carbon stocks over six decades: A chronosequence study. Journal of Soils and Sediments 23, 1193–1203.

[r128] UNEP (2020) Guidelines for Seagrass Ecosystem Restoration in the Western Indian Ocean Region. Nairobi, Kenya.

[r129] UNEP-WCMC (2022) Progress, Needs and Opportunities for Seascape Restoration. Cambridge, UK.

[r130] Valle M, Borja Á, Chust G, Galparsoro I and Garmendia JM (2011) Modelling suitable estuarine habitats for Zostera noltii, using ecological niche factor analysis and bathymetric LiDAR. Estuarine, Coastal and Shelf Science 94, 144–154.

[r131] Valle M, Garmendi JM, Chust G, Franco J and Borja Á (2015) Increasing the chance of a successful restoration of Zostera noltii meadows. Aquatic Botany 127, 12–19.

[r132] van Katwijk MM, Bos AR, de Jonge VN, Hanssen LSAM, Hermus DCR and de Jong DJ (2009) Guidelines for seagrass restoration: Importance of habitat selection and donor population, spreading of risks, and ecosystem engineering effects. Marine Pollution Bulletin 58, 179–188. 19131078 10.1016/j.marpolbul.2008.09.028

[r133] van Katwijk MM, Thorhaug A, Marbà N, Orth RJ, Duarte CM, Kendrick GA, Althuizen IHJ, Balestri E, Bernard G, Cambridge ML, Cunha A, Durance C, Giesen W, Han Q, Hosokawa S, Kiswara W, Komatsu T, Lardicci C, Lee K, Meinesz A, Nakaoka M, O’Brien KR, Paling EI, Pickerell C, Ransijn AMA and Verduin JJ (2016) Global analysis of seagrass restoration: The importance of large‐scale planting. Journal of Applied Ecology 53, 567–578.

[r134] van Regteren M, Meesters EH, Baptist MJ, De Groot AV, Bouma TJ and Elschot K (2020) Multiple environmental variables affect germination and mortality of an annual salt marsh pioneer: *Salicornia procumbens*. Estuaries and Coasts 43, 1489–1501.

[r135] Walcker R, Gandois L, Proisy C, Corenblit D, Mougin É, Laplanche C, Ray R and and Fromard F (2018) Control of “blue carbon” storage by mangrove ageing: Evidence from a 66-year chronosequence in French Guiana. Global Change Biology 24, 2325–2338. 29474752 10.1111/gcb.14100

[r136] Waltham NJ, Elliott M, Lee SY, Lovelock C, Duarte CM, Buelow C, Simenstad C, Nagelkerken I, Claassens L and Wen CK (2020) UN decade on ecosystem restoration 2021–2030—What chance for success in restoring coastal ecosystems? Frontiers in Marine Science 7, 71.

[r137] Waltham NJ, Alcott C, Barbeau MA, Cebrian J, Connolly RM, Deegan LA, Dodds K, Goodridge Gaines LA, Gilby BL, Henderson CJ, McLuckie CM, Minello TJ, Norris GS, Ollerhead J, Pahl J, Reinhardt JF, Rezek RJ, Simenstad CA, Smith JAM, Sparks EL, Staver LW, Ziegler SL and Weinstein MP (2021) Tidal marsh restoration optimism in a changing climate and urbanizing seascape. Estuaries and Coasts 44, 1681–1690.

[r138] Wang G, Yu C, Singh M, Guan D, Xiong Y, Zheng R and Xiao R (2021) Community structure and ecosystem carbon stock dynamics along a chronosequence of mangrove plantations in China. Plant and Soil 464, 605–620.

[r139] Ward M and Beheshti K (2023) Lessons learned from over thirty years of eelgrass restoration on the US West coast. Ecosphere 14, e4642.

[r140] Williams SL, Ambo-Rappe R, Sur C, Abbott JM and Limbong SR (2017) Species richness accelerates marine ecosystem restoration in the coral triangle. Proceedings of the National Academy of Sciences 114, 11986–11991. 10.1073/pnas.1707962114 PMC5692552 29078320

[r141] Wodehouse DCJ and Rayment MB (2019) Mangrove area and propagule number planting targets produce sub-optimal rehabilitation and afforestation outcomes. Estuarine, Coastal and Shelf Science 222, 91–102.

[r142] Wolters M, Garbutt A and Bakker JP (2005) Salt-marsh restoration: Evaluating the success of de-embankments in North-West Europe. Biological Conservation 123, 249–268.

[r143] Worthington TA and Spalding M (2018) Mangrove Restoration Potential: A Global Map Highlighting a Critical Opportunity. Cambridge: University of Cambridge.

[r144] Worthington TA, Spalding M, Landis E, Maxwell TL, Navarro A, Smart LS and Murray NJ (2024) The distribution of global tidal marshes from earth observation data. *Global Ecology and Biogeography*, e13852

[r145] Worthington TA, zu Ermgassen PSE, Friess DA, Krauss KW, Lovelock CE, Thorley J, Tingey R, Woodroffe CD, Bunting P, Cormier N, Lagomasino D, Lucas R, Murray NJ, Sutherland WJ and Spalding M (2020) A global biophysical typology of mangroves and its relevance for ecosystem structure and deforestation. Scientific Reports 10, 14652. 32887898 10.1038/s41598-020-71194-5 PMC7473852

[r146] Zeng Y, Sarira TV, Carrasco LR, Chong KY, Friess DA, Lee JSH, Taillardat P, Worthington TA, Zhang Y and Koh LP (2020) Economic and social constraints on reforestation for climate mitigation in Southeast Asia. Nature Climate Change 10, 842–844.

[r147] Zhang J-P, Shen C-D, Ren H, Wang J and Han W-D (2012) Estimating change in sedimentary organic carbon content during mangrove restoration in Southern China using carbon isotopic measurements. Pedosphere 22, 58–66.

[r148] Zimmer M, Ajonina GN, Amir AA, Cragg SM, Crooks S, Dahdouh-Guebas F, et al. (2022) When nature needs a helping hand: Different levels of human intervention for mangrove (re-)establishment. Frontiers in Forests and Global Change 5, 784322.

