# Supplementary material for: Restoring blue carbon ecosystems
Source: Camb Prism Coast Futur. 2024 May 17;2:e9. doi: 10.1017/cft.2024.9 (PMC12337601; doi:10.1017/cft.2024.9)
Supplement: Peer review history

# Editor report: Recommendation: Restoring blue carbon ecosystems — R0/PR2

**DOI:** 10.1017/cft.2024.9.pr2

**Affiliations:** Sun Yat-Sen University, China

Dear authors,

On behalf of the editorial board, I would like to inform you a minor revision decision on your manuscript had been recommended. Please refer to the comments provided by two reviewers which I believed to be helpful to improve your works effectively. Many thanks!

Yisheng PENG

# Editor report: Decision: Restoring blue carbon ecosystems — R0/PR3

**DOI:** 10.1017/cft.2024.9.pr3

**Affiliations:** Geography, Cambridge University, United Kingdom of Great Britain and Northern Ireland

No accompanying comment.

# Editor report: Recommendation: Restoring blue carbon ecosystems — R1/PR5

**DOI:** 10.1017/cft.2024.9.pr5

**Affiliations:** Sun Yat-Sen University, China

Dear Dr Friess,

I am pleased to inform you that your submission entitiled “Restoring blue carbon systems” has been accepted for publication in Cambridge Prisms: Costal Futures. Thank you for giving us the opportunity to review your article.

Sincerely,

Yisheng Peng

# Editor report: Decision: Restoring blue carbon ecosystems — R1/PR6

**DOI:** 10.1017/cft.2024.9.pr6

**Affiliations:** Geography, Cambridge University, United Kingdom of Great Britain and Northern Ireland

No accompanying comment.
